# Genetic Variation of Flavonols Quercetin, Myricetin, and Kaempferol in the Sri Lankan Tea (*Camellia sinensis* L.) and Their Health-Promoting Aspects

**DOI:** 10.1155/2016/6057434

**Published:** 2016-06-06

**Authors:** Brasathe Jeganathan, P. A. Nimal Punyasiri, J. Dananjaya Kottawa-Arachchi, Mahasen A. B. Ranatunga, I. Sarath B. Abeysinghe, M. T. Kumudini Gunasekare, B. M. Ratnayake Bandara

**Affiliations:** ^1^Department of Food Science and Technology, Faculty of Agriculture, University of Peradeniya, Peradeniya, Sri Lanka; ^2^Institute of Biochemistry Molecular Biology and Biotechnology, University of Colombo, 00300 Colombo, Sri Lanka; ^3^Tea Research Institute of Sri Lanka, Talawakelle, Sri Lanka; ^4^Coordinating Secretariat for Science, Technology & Innovation, 3rd Floor, Standard Charted Building, Janadhipathi Mawatha, 00100 Colombo, Sri Lanka; ^5^Department of Chemistry, Faculty of Science, University of Peradeniya, Peradeniya, Sri Lanka

## Abstract

Flavonol glycosides in tea leaves have been quantified as aglycones, quercetin, myricetin, and kaempferol. Occurrence of the said compounds was reported in fruits and vegetable for a long time in association with the antioxidant potential. However, data on flavonols in tea were scanty and, hence, this study aims to envisage the flavonol content in a representative pool of accessions present in the Sri Lankan tea germplasm. Significant amounts of myricetin, quercetin, and kaempferol have been detected in the beverage type tea accessions of the Sri Lankan tea germplasm. This study also revealed that tea is a good source of flavonol glycosides. The* Camellia sinensis* var.* sinensis* showed higher content of myricetin, quercetin, and total flavonols than var.* assamica* and ssp.* lasiocalyx*. Therefore flavonols and their glycosides can potentially be used in chemotaxonomic studies of tea germplasm. The nonbeverage type cultivars, especially* Camellia rosaflora* and* Camellia japonica* Red along with the exotic accessions resembling China type, could be useful in future germplasm studies because they are rich sources of flavonols, namely, quercetin and kaempferol, which are potent antioxidants. The flavonol profiles can be effectively used in choosing parents in tea breeding programmes to generate progenies with a wide range of flavonol glycosides.

## 1. Introduction

Tea is the second most popular beverage after water which represents a major source of dietary polyphenols [1]. Thus the scientific community is interested in exploring the health-promoting constituents present in tea, namely, flavan-3-ols, flavonols, and their derivatives [2]. Recent epidemiological studies have demonstrated a protective effect of fruits and vegetables against the incidence of degenerative diseases [3]. Several classes of compounds have been assumed as potential protective factors, one of which is the flavonoids, generally considered as nonnutritive agents. Flavonoids are known as secondary metabolites which are the largest class of polyphenols widely distributed in the plant kingdom. It can be subdivided into six major subclasses based on the structural variations: flavones, flavanones, isoflavones, flavonols, flavanols, and anthocyanidins [4].

According to the study conducted by Hollman et al. [5] tea is a good source of flavonols. Flavonols have recently received much attention due to their antioxidant, antimicrobial, anticancer, antiatherosclerotic, and antiproliferative properties [6–8]. The flavonols exist in tea plant (*Camellia sinensis* L.) as flavonol glycosides with a sugar residue and the most common flavonol aglycones in tea are quercetin, kaempferol, and myricetin [9].

Ceylon tea from Sri Lanka, acclaimed as the best tea in the world, has its inherent unique characteristics and reputation running over more than a century [10]. The objectives of this study were to investigate the variation in flavonol aglycones, namely, quercetin, kaempferol, and myricetin in tea (*Camellia sinensis* L.) accessions including beverage and nonbeverage types. Therefore, we report here the first systematic study on the flavonol content in representative accessions of the Sri Lankan tea germplasm.

## 2. Materials and Methods

### 2.1. Collection and Preparation of Tea Samples for Analysis

50 g of tender tea shoots (two apical leaves and the bud) of 87 beverage type and 6 nonbeverage type germplasm accessions was collected in triplicate from the* ex situ* field gene bank of Tea Research Institute of Sri Lanka (TRI), Talawakelle (latitude 6°54′N, longitude 80°42′E), and brought to the laboratory at 4°C. Selection criteria for germplasm accessions in this study included the attributes such as morphological variations, origins, made tea quality, and sensitivity to biotic stresses [11–13]. The tea samples were immediately stored in a −80°C freezer for 6 h to two weeks and freeze-dried for 24 h (Labconco Corporation, MS, USA). The freeze-dried leaves were ground to a fine powder (passed through 500 *μ*m mesh), sealed in triple laminated aluminium foil packages, and stored at 20°C until biochemical analysis [14].

### 2.2. Extraction of Flavonols

100 mg of freeze-dried tea sample was hydrolysed in duplicate with 0.5 mL of 6 M HCl and 4.5 mL of 60% methanol for 2 h in a water bath preheated to 95°C. Hydrolysed sample extracts were topped up to 5 mL with 60% methanol and filtered through 0.45 *μ*m nylon filters into HPLC vials.

### 2.3. Preparation of Standards and Calibration Curves

Standards of myricetin, quercetin, and kaempferol (Sigma, St. Louis, USA) were prepared in 60% methanol. Calibration curves were constructed for the individual compounds. Peak identity was also confirmed by the photodiode array UV spectra.

### 2.4. Quantification of Flavonols

**Figure 1:**
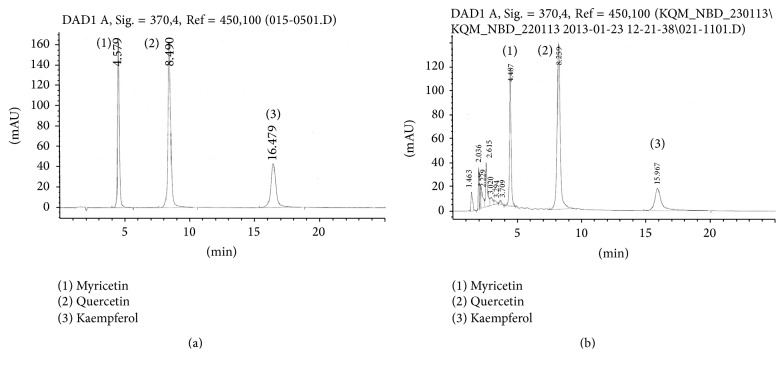
(a) HPLC chromatogram of mixed standard solution comprising kaempferol, quercetin, and myricetin. (b) Representative tea accession quantified and resolved by HPLC.

Analysis of individual flavonols was carried out in the Agilent 1260 Infinity HPLC system comprising of solvent delivery system, quaternary pump, temperature-controlled column oven, autosampler, degasser unit and a photodiode array detector with UV detection at 370 nm, and Open Lab/Chemstation Software system. The compounds were separated on a 4.6 × 250 mm, 5 *μ*m particle, and reversed phase ZORBAX Eclipse Plus C 18 column (Agilent, USA) (Phenomenex Inc., USA) with a guard column made of the same material (Security-Guard, Phenomenex Inc., USA) at 30°C. 30% HPLC grade acetonitrile in 0.025 M potassium dihydrogen orthophosphate buffer solution adjusted to a pH of 2.5 with 6 M HCl was used as mobile phase at a flow rate of 1.0 mL/min. The injection volume was 10 *μ*L. Quantification was carried out from the integrated peak areas of the sample against the corresponding standard graph and results were expressed on dry matter basis (Figures 1(a) and 1(b)). The dry matter contents of the freeze-dried leaves were determined in accordance with ISO1573:1980.

### 2.5. Statistical Analysis

The results were expressed as mean ± SD of triplicate data. Experimental data were analysed using SAS system for Windows V 9.1 (SAS Institute Inc., NC, USA).

### 2.6. Reagents

Absolute methanol (analytical reagent grade), anhydrous sodium carbonate, and acetonitrile (HPLC gradient grade) were supplied by Fisher Scientific UK; quercetin, kaempferol, and myricetin were purchased from Sigma Chemicals; and potassium dihydrogen orthophosphate and hydrochloric acid (analytical grade) were purchased from British Drug House Ltd., England.

## 3. Results and Discussion

### 3.1. Flavonols Content of Beverage and Nonbeverage Type Accessions

The major flavonols present in tea leaves make up 2-3% of the water-soluble solids in tea [15, 16].  Table 1 shows the amounts of myricetin, quercetin, and kaempferol quantified in the beverage and nonbeverage type accessions of the Sri Lankan tea germplasm. These results are agreeable to a greater extend with the results reported for 3 commercial samples of green tea and 30 commercial samples of black tea [17] and those reported for 4 commercial samples of green tea and 2 commercial samples of black tea [18]. According to the mean values of flavonols, quercetin (1.50 mg g^−1^) is abundantly present in the beverage type accessions of the Sri Lankan tea germplasm followed by kaempferol (1.31 mg g^−1^) and myricetin (0.94 mg g^−1^). The mean value of total flavonols in 89 beverage type* C. sinensis* was 3.75 mg g^−1^.

Among beverage type accessions, the highest quercetin contents were reported in the exotic accessions: PBGT41 (3.23 mg g^−1^), followed by PBGT73, China, PBGT70, and PBGT12 whereas TRI1114 showed the lowest content (0.36 mg g^−1^). The highest kaempferol contents were observed in the exotic accessions PBGT61 (2.21 mg g^−1^) followed by PBGT68, PBGT48, PBGT49, and PBGT70. Among the 9 exotic accessions examined, PBGT41 reported the highest myricetin content (1.63 mg g^−1^) followed by PBGT49, PBGT68, PBGT70, and PBGT61 and INTRI6 recorded the lowest content (0.33 mg g^−1^).

Conversely, myricetin was available in trace amounts recorded in the nonbeverage type accessions except* Camellia sasanqua *(0.37 mg g^−1^) and* Camellia rosaflora* (0.20 mg g^−1^). Highest kaempferol contents were observed in* Camellia japonica donkelarri *and* Camellia japonica *Red.* Camellia rosaflora* has reported the highest quercetin content (3.44 mg g^−1^) when considering the entire germplasm, which is higher than that of the beverage type exotic cultivar PBGT41. This special characteristic can be utilized in future germplasm studies.

The specific patterns of flavonol occurrence in plant species vary based on their synthesis and distribution in the plant organism [19]. In addition to intrinsic factors, flavonol content is strongly influenced by extrinsic factors such as variations in plant type and growth, season, climate, degree of maturity, preparation, and processing [20–24].

Several studies report varying content of flavonol in teas [1, 25, 26]. This is possibly due to the difference in tea samples used (fresh tea leaves or processed tea) and analytical methods utilized, resulting in different extraction efficiencies. According to Finger et al. [27], flavonol glycosides are not affected by polyphenol oxidase and varying content in different tea types can be due to difference in tea varieties, geographic location, or agricultural conditions.

### 3.2. Health Aspects of Tea Flavonols

The increasing knowledge on functional ingredients in food provides new opportunities for plant sciences to produce crops with higher amounts of selected health-promoting phytochemicals using crop husbandry techniques, plant breeding techniques, and genetic engineering [28]. Recent studies suggest that higher intakes of flavonoids from food are associated with reduced risk of degenerative diseases including cancer and heart disease [29, 30].

The three flavonols, that is, myricetin, quercetin, and kaempferol, quantified in this study have also been extensively investigated for their health benefits. Flavonols naturally occur as either glycosides (with attached glycosyl groups) or aglycones. However, dietary intake of the above flavonols is mainly in the glycoside form. During digestion and absorption sugar moieties can be released from the glycosides. Digestive tract microbiota could also assist in the digestion and the absorption process of the flavonols. Following absorption several organs contribute to flavonol metabolism including the liver, giving rise to glucuronidated, methylated, and sulfated forms of flavonols which are found in the plasma. Further, free flavonols (aglycone) are also found in the plasma. These circulating flavonols and their metabolites have diverse biological activities [31].

Several mechanisms of actions such as antioxidant activity, anti-inflammatory activity, and modulation of signaling pathways are responsible for the beneficial effects of flavonols [32].

Prolonged oxidative stress could lead to several degenerative diseases including cardiovascular disease. Ability of hydrogen radical donation from the phenolic group and the presence of an unpaired electron in the aromatic ring make the flavonols strong antioxidants* in vivo*, thus scavenging the reactive oxygen species and other free radicals. Ability to chelate metal ions also reduces the production of harmful oxidant species* in vivo* [33]. Flavonols could contribute to the antioxidant properties through the protection or improvement of endogenous antioxidants especially by stimulating glutathione-*S*-transferase (GST), an enzyme that protects cells from the damage caused by free radicals [34].

The inflammatory response is part of the innate immune response which triggers the inflammatory cascade forming inflammatory mediators such as prostaglandin and thromboxane. Although acute inflammation is essential in dealing with invading pathogens and injury, chronic inflammation can cause tissue destruction and is involved in the pathogenesis of degenerative diseases. Commercially available anti-inflammatory drugs are used to prevent the formation of prostaglandins and thromboxanes by inhibiting the cyclooxygenase (COX) enzymes involved in the production of the above during inflammation. Flavonoids can suppress the expression of the COX gene through interactions with cell signaling pathways, such as the protein kinase C, NF-*κ*B, and tyrosine kinase pathways [35].

It has been reported that flavonols including myricetin, quercetin, and kaempferol could play an important role in reducing the risk of cardiovascular disease, cancer, and neurodegradative and other degenerative diseases through the above-mentioned mechanisms and several other mechanisms. Several epidemiological studies also have found the beneficial effects of flavonols [36, 37]. Therefore, it could be argued that dietary items high in flavonols could be beneficial to human health.

Although flavonol glycosides can be important contributors to black tea flavour [38], these were hydrolysed by acid treatment to afford myricetin, quercetin, and kaempferol for HPLC analysis due to the fact that the dietary forms of flavonol glycosides are initially hydrolysed to aglycones at the oral cavity [39–42] and continued in the digestive tract [5, 43]. Even during the fermentation processes the flavonoid glycosides undergo enzymatic hydrolysis and the level of flavonoid aglycones increase. However the chemical form of flavonol prior to and after digestion is of great importance in determining the bioavailability and bioactivity.

### 3.3. Utilization of Flavonol Diversity in Tea Breeding

In a single plant very large number of individual glycosides may result based on the type and number of sugar residues, together with chain branching. Some of these flavonols were quite useful for discriminating genotypes in different species. Vrhovsek et al. [44] reported that myricetin glycosides had some significant level of variability among the blueberry cultivars. This means that both the biosynthesis of the different aglycones and their conjugation with different sugars are controlled by the genotype. Another comprehensive survey of flavonol content in 100 vegetables and fruits grown in China reported that quercetin was widely distributed in edible plants species [45].

Flavonol quercetin was the main compound found in many fruits and vegetables [46]. de Vries et al. [47] explored the bioavailability of quercetin from red wine, yellow onions, and black tea and came to the conclusion that tea is a rich source of flavonols. Quercetin and kaempferol rhamnodiglucosides are characteristic compounds of* Camellia sinensis*. According to Finger et al. [27], black tea contains 0–0.95 mg g^−1^ quercetin rhamnodiglucoside and 0.05–1.25 mg g^−1^ kaempferol rhamnodiglucoside. However myricetin and kaempferol were also detected in many foods including tea [1, 46, 48, 49].

Based on the records available, less than 6% of the total accessions preserved in Sri Lankan tea germplasm have been utilized in the tea breeding programmes till 1998, and utilization of tea accessions has increased up to 13.6% by the end of 2009 [50]. The lack of information regarding the genetic variation, mainly biochemical and molecular diversity of the existing population, could be one reason for underutilization of the germplasm [12].

**Figure 2:**
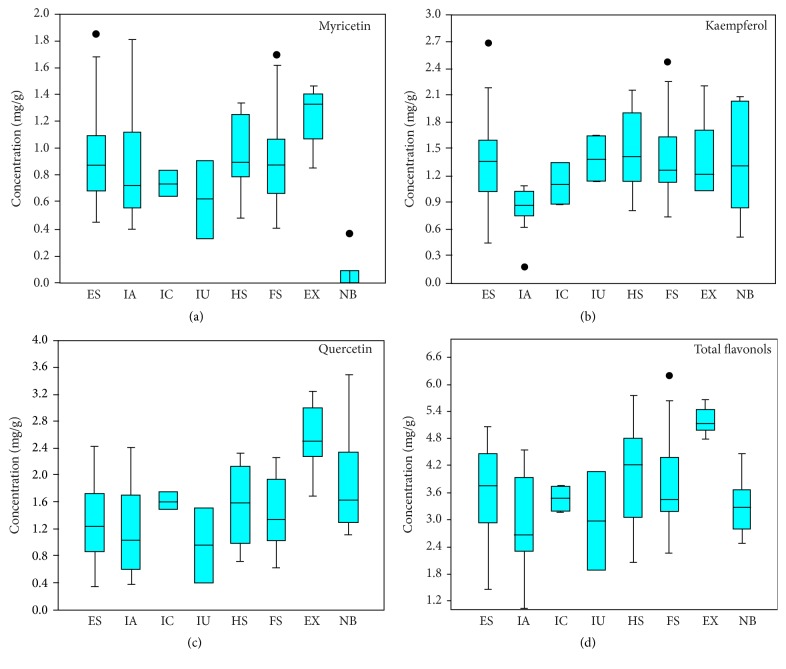
Distribution of total and individual flavonols in cultivated* Camellia sinensis* varieties and nonbeverage types (ES: estate selections; IA: introductions from Assam; IC: introductions from Indo-China; IU: unknown introductions; HS: half-sib families; FS: full-sib families; EX: exotic varieties; NB: nonbeverage types; and ● indicates outliers).

According to present study, the lowest mean value of kaempferol content is observed for Assam introductions (Figures 2(a)–2(d)). The ranges and mean values of quercetin and myricetin for full-sib and half-sib families are much similar to the Assam introductions. Therefore, it is indicated that the TRI developed accessions have direct or indirect relationship with Assam introductions. Among the selected accessions, all exotic accessions recorded higher contents of quercetin than rest. Besides, the highest mean values of total flavonol contents were reported for exotic accessions. These results revealed that those exotic accessions are having different geographic origin than other accessions present in germplasm in Sri Lanka.

### 3.4. Variations in Flavonols among Varieties of* C. sinensis*

Average values of the flavonols of China type (var.* sinensis*), Assam type (var.* assamica*), and Cambod type (ssp.* lasiocalyx*) of* C. sinensis* (L.) are given in Table 2. Flavonols, myricetin and quercetin, contents of the var.* sinensis* were higher than that of the var.* assamica* and ssp.* lasiocalyx*. Beside, var.* assamica* had the highest kaempferol content than other varieties and nonbeverage types. Among all three varieties and nonbeverage types, var.* sinensis* showed the highest total flavonols. Sri Lankan germplasm collection was predominantly represented by ssp.* lasiocalyx* followed by* assamica* and* sinensis *varieties. Around 83% of the introductions acquired prior to the 1960s, mainly from India and Indo-China, exhibit predominant Cambod type (ssp.* lasiocalyx*) characters and introductions made in 21st century from Korea reminiscent of more affinity to China type (var.* sinensis*). However, China type accessions are inadequately represented in the collection [51]. Therefore, flavonols and their glycosides can be useful to chemotaxonomic studies of tea germplasm.

As evident from the present work, the 9 exotic accessions with different geographic origin selected for this study along with the nonbeverage type accessions, especially* Camellia rosaflora* and* Camellia japonica *Red, are worthwhile and thus merit focus in future germplasm studies. The contents of myricetin and myricetin glycosides are less in black tea; unlikely, in fresh tea, they flush since they can still be affected by tea processing. Myricetin and myricetin glycosides are the most likely of the three flavonols to be oxidised [52]. However the presence of myricetin is higher in tea compared to other herbal plants [18]. Therefore it is crucial to focus on myricetin while discussing the flavonol content of tea.

## 4. Conclusions

Significant amounts of myricetin, quercetin, and kaempferol have been quantified in the beverage type tea accessions of the Sri Lankan tea germplasm. The var.* sinensis* showed higher content of myricetin, quercetin, and total flavonol than var.* assamica* and ssp.* Lasiocalyx*; therefore flavonols and their glycosides can be useful to chemotaxonomic studies of tea germplasm. The nonbeverage type cultivars, especially* Camellia rosaflora* and* Camellia japonica* Red along with the exotic accessions resembling China type, are worthwhile in future germplasm studies because they are rich sources of flavonols, namely, quercetin and kaempferol, which are potent antioxidants and health-promoting aspects. Additionally, the flavonol profiles can be effectively used in choosing accessions in tea breeding programmes to generate progenies with wide variations.

## Figures and Tables

**Table 1:** Flavonol content of Sri Lankan tea accessions (all units are in mg g^−1^; data represent the mean of six replicates ± standard error).

Accessions		Myricetin (mg g^−1^)	Quercetin (mg g^−1^)	Kaempferol (mg g^−1^)	Total flavonols (mg g^−1^)
	*Estate selections*				
1	B275	0.54 ± 0.08	0.85 ± 0.06	0.90 ± 0.06	2.29 ± 0.19
2	CH13	0.92 ± 0.10	1.11 ± 0.11	2.19 ± 0.12	4.22 ± 0.69
3	CV4B1	0.56 ± 0.06	0.91 ± 0.08	1.58 ± 0.23	3.05 ± 0.52
4	CY9	1.68 ± 0.17	1.69 ± 0.11	1.70 ± 0.16	5.07 ± 0.01
5	DEL40	0.46 ± 0.02	1.37 ± 0.01	1.48 ± 0.06	3.31 ± 0.56
6	DG39	1.08 ± 0.12	2.09 ± 0.11	1.34 ± 0.18	4.51 ± 0.53
7	DG7	0.69 ± 0.18	0.43 ± 0.04	1.05 ± 0.09	2.17 ± 0.31
8	DK24	0.75 ± 0.01	2.42 ± 0.11	1.33 ± 0.09	4.50 ± 0.85
9	DN	0.85 ± 0.32	1.17 ± 0.30	0.73 ± 0.11	2.75 ± 0.23
10	DT1	1.09 ± 0.04	2.04 ± 0.18	0.60 ± 0.05	3.74 ± 0.73
11	DT95	0.88 ± 0.11	0.92 ± 0.12	2.69 ± 0.09	4.49 ± 1.03
12	DUN7	0.99 ± 0.06	1.90 ± 0.52	1.43 ± 0.08	4.33 ± 0.46
13	H1/58	0.45 ± 0.04	0.71 ± 0.04	1.24 ± 0.15	2.41 ± 0.40
14	HS10A	0.81 ± 0.11	1.07 ± 0.02	1.51 ± 0.29	3.39 ± 0.35
15	K145	1.08 ± 0.07	1.29 ± 0.51	1.48 ± 0.79	3.86 ± 0.20
16	KEN16/3	0.92 ± 0.08	0.78 ± 0.16	1.46 ± 0.15	3.16 ± 0.36
17	KP204	1.22 ± 0.17	2.43 ± 0.07	0.84 ± 0.10	4.50 ± 0.83
18	MO241	0.60 ± 0.03	1.02 ± 0.13	1.47 ± 0.10	3.08 ± 0.44
19	MT18	1.85 ± 0.95	1.76 ± 0.04	1.19 ± 0.04	4.81 ± 0.36
20	N2	1.12 ± 0.12	1.69 ± 0.09	1.06 ± 0.21	3.87 ± 0.35
21	NAY3	1.52 ± 0.05	2.01 ± 0.05	1.22 ± 0.10	4.76 ± 0.40
22	NL8/3	1.35 ± 0.08	1.71 ± 0.07	0.73 ± 0.28	3.79 ± 0.50
23	PK2	1.31 ± 0.18	1.96 ± 0.17	0.98 ± 0.15	4.25 ± 0.49
24	PLLG2	0.74 ± 0.11	1.06 ± 0.12	1.92 ± 0.03	3.72 ± 0.61
25	PO26	0.50 ± 0.07	0.63 ± 0.02	1.36 ± 0.10	2.48 ± 0.46
26	QT4/4	1.10 ± 0.03	1.14 ± 0.03	1.46 ± 0.02	3.69 ± 0.20
27	S106	0.91 ± 0.01	0.73 ± 0.03	1.60 ± 0.02	3.24 ± 0.46
28	TC10	0.86 ± 0.07	1.75 ± 0.09	1.73 ± 0.08	4.34 ± 0.51
29	TC9	1.48 ± 0.14	1.68 ± 0.18	1.87 ± 0.02	5.03 ± 0.19
30	TRI1114	0.64 ± 0.06	0.36 ± 0.10	0.45 ± 0.03	1.45 ± 0.14
31	TRI1294	0.93 ± 0.07	1.40 ± 0.25	2.10 ± 0.44	4.43 ± 0.59
32	TRI2142	0.78 ± 0.04	1.67 ± 0.22	1.34 ± 0.23	3.79 ± 0.45
33	TRI26	1.06 ± 0.06	1.53 ± 0.15	2.14 ± 0.29	4.73 ± 0.54
34	TRI3011	0.67 ± 0.13	1.25 ± 0.07	0.69 ± 0.13	2.60 ± 0.33
35	W3	0.79 ± 0.10	0.98 ± 0.22	1.46 ± 0.21	3.23 ± 0.35
36	WT26	0.70 ± 0.18	0.85 ± 0.02	1.26 ± 0.12	2.81 ± 0.29
37	TRI2016	0.61 ± 0.08	0.55 ± 0.07	0.67 ± 0.09	1.84 ± 0.06

	*Introductions from Assam*				
38	ASM4/10	0.76 ± 0.06	1.08 ± 0.13	1.01 ± 0.06	2.85 ± 0.17
39	TRI2022	1.81 ± 0.10	2.12 ± 0.53	0.63 ± 0.10	4.56 ± 0.79
40	TRI2023	1.35 ± 0.05	1.59 ± 0.26	0.79 ± 0.09	3.74 ± 0.41
41	TRI2024	0.68 ± 0.08	0.63 ± 0.05	1.09 ± 0.20	2.40 ± 0.25
42	TRI2025	0.40 ± 0.04	0.59 ± 0.15	0.99 ± 0.06	1.99 ± 0.30
43	TRI2026	0.79 ± 0.17	0.94 ± 0.07	0.87 ± 0.11	2.60 ± 0.08
44	TRI3047	0.59 ± 0.02	1.30 ± 0.59	0.83 ± 0.14	2.73 ± 0.36
45	TRI3055	1.05 ± 0.06	2.42 ± 0.15	1.07 ± 0.06	4.54 ± 0.79
46	TRI62/5	0.67 ± 0.06	1.03 ± 0.01	0.87 ± 0.16	2.57 ± 0.18
47	TRI62/9	0.44 ± 0.04	0.40 ± 0.05	0.18 ± 0.03	1.02 ± 0.14

	*Introductions from Indo-China*				
48	TRI777	0.65 ± 0.05	1.75 ± 0.13	1.34 ± 0.23	3.74 ± 0.56
49	TRI2043	0.83 ± 0.05	1.50 ± 0.64	0.89 ± 0.02	3.21 ± 0.38

	*Unknown introductions*				
50	INTRI6	0.33 ± 0.04	0.41 ± 0.07	1.14 ± 0.13	1.87 ± 0.45
51	VHMOR	0.91 ± 0.15	1.52 ± 0.07	1.64 ± 0.21	4.07 ± 0.39

	*Half-sib family*				
52	TRI3013	0.48 ± 0.04	0.75 ± 0.11	0.81 ± 0.12	2.05 ± 0.18
53	TRI3014	0.82 ± 0.04	2.22 ± 0.03	1.84 ± 0.04	4.88 ± 0.72
54	TRI3015	1.16 ± 0.08	1.64 ± 0.05	1.39 ± 0.03	4.19 ± 0.24
55	TRI3041	1.28 ± 0.06	2.33 ± 0.36	2.16 ± 0.53	5.76 ± 0.56
56	TRI3050	1.34 ± 0.10	1.84 ± 0.12	1.44 ± 0.02	4.61 ± 0.26
57	TRI3072	0.88 ± 0.09	1.51 ± 0.25	1.30 ± 0.07	3.69 ± 0.32
58	TRI3073	0.79 ± 0.08	1.51 ± 0.21	1.92 ± 0.38	4.22 ± 0.57
59	TRI3045	0.92 ± 0.18	0.84 ± 0.12	1.08 ± 0.19	2.84 ± 0.13

	*Full-sib family*				
60	TRI3016	0.59 ± 0.06	2.15 ± 0.18	1.67 ± 0.25	4.41 ± 0.80
61	TRI3017	0.96 ± 0.06	2.04 ± 0.14	2.47 ± 0.33	5.47 ± 0.78
62	TRI3018	0.81 ± 0.09	1.71 ± 0.02	1.16 ± 0.10	3.68 ± 0.45
63	TRI3019	1.09 ± 0.10	1.92 ± 0.28	1.13 ± 0.07	4.15 ± 0.47
64	TRI3026	0.63 ± 0.02	0.81 ± 0.06	0.83 ± 0.07	2.27 ± 0.11
65	TRI3036	0.40 ± 0.05	0.89 ± 0.21	1.20 ± 0.16	2.48 ± 0.40
66	TRI4004	0.93 ± 0.10	2.00 ± 0.04	2.26 ± 0.03	5.18 ± 0.71
67	TRI4049	0.84 ± 0.07	1.23 ± 0.27	1.39 ± 0.13	3.45 ± 0.28
68	TRI4052	0.78 ± 0.12	1.14 ± 0.06	1.47 ± 0.20	3.39 ± 0.35
69	TRI4053	0.45 ± 0.03	0.94 ± 0.11	0.93 ± 0.10	2.32 ± 0.28
70	TRI4061	1.70 ± 0.05	2.27 ± 0.31	2.24 ± 0.12	6.21 ± 0.32
71	TRI4063	0.63 ± 0.04	1.42 ± 0.04	1.27 ± 0.22	3.32 ± 0.42
72	TRI4067	0.98 ± 0.19	1.02 ± 0.22	1.26 ± 0.07	3.26 ± 0.15
73	TRI4068	0.78 ± 0.05	0.64 ± 0.18	1.13 ± 0.10	2.55 ± 0.26
74	TRI4071	0.76 ± 0.12	1.10 ± 0.17	1.30 ± 0.13	3.16 ± 0.28
75	TRI4076	1.62 ± 0.07	1.91 ± 0.12	0.74 ± 0.08	4.27 ± 0.61
76	TRI4078	1.00 ± 0.08	1.33 ± 0.16	1.55 ± 0.04	3.88 ± 0.28
77	TRI4079	0.91 ± 0.07	1.37 ± 0.08	1.16 ± 0.06	3.44 ± 0.23
78	TRI4085	1.27 ± 0.09	1.15 ± 0.24	0.90 ± 0.06	3.31 ± 0.19
79	TRI3022	1.48 ± 0.05	1.95 ± 0.06	2.19 ± 0.11	5.63 ± 0.36

	*Exotics types*				
80	China	0.85 ± 0.10	2.98 ± 0.21	1.02 ± 0.12	4.85 ± 1.19
81	PBGT61	1.34 ± 0.02	1.70 ± 0.03	2.21 ± 0.17	5.24 ± 0.44
82	PBGT68	1.42 ± 0.08	2.39 ± 0.07	1.63 ± 0.06	5.44 ± 0.51
83	PBGT48	1.31 ± 0.13	2.34 ± 0.10	1.41 ± 0.05	5.06 ± 0.57
84	PBGT49	1.46 ± 0.14	2.41 ± 0.08	1.23 ± 0.04	5.10 ± 0.63
85	PBGT12	1.15 ± 0.83	2.61 ± 0.28	1.05 ± 0.15	4.81 ± 0.88
86	PBGT41	1.40 ± 0.56	3.23 ± 0.39	1.03 ± 0.08	5.66 ± 1.17
87	PBGT70	1.39 ± 0.15	2.85 ± 0.15	1.21 ± 0.03	5.45 ± 0.90
88	PBGT73	0.96 ± 0.07	3.05 ± 0.04	1.02 ± 0.09	5.03 ± 1.19
89	Yabukita	1.11 ± 0.17	2.12 ± 0.03	1.94 ± 0.09	5.17 ± 0.54
	*Mean* (*n* = 89)	*0.94*	*1.50*	*1.31*	*3.75*

	*Nonbeverage types*				
90	*C. sasanqua*	0.37 ± 0.05	1.59 ± 0.17	0.52 ± 0.03	2.48 ± 0.67
91	*C. japonica donkelarri*	ND	1.13 ± 0.02	2.08 ± 0.06	3.21 ± 1.04
92	*C. japonica herculea*	ND	1.96 ± 0.18	0.95 ± 0.09	2.91 ± 0.98
93	*C. japonica *Red	ND	1.36 ± 0.07	2.02 ± 0.05	3.38 ± 1.03
94	*C. japonica *Whit*e*	ND	1.70 ± 0.07	1.60 ± 0.06	3.31 ± 0.96
95	*C. rosaflora*	0.20 ± 0.00	3.44 ± 0.03	1.03 ± 0.08	4.67 ± 1.77
	*Mean* (*n* = 6)	*0.28*	*1.86*	*1.37*	*3.33*

ND: not detected.

**Table 2:** Flavonols profile of three varieties of *C*. *sinensis* (all units are in mg g^−1^; data represent the mean of six replicates ± standard error).

Compounds	var. *sinensis* (*n* = 10)	var. *assamica* (*n* = 19)	ssp. *lasiocalyx* (*n* = 60)	Nonbeverage types (*n* = 6)
Myricetin	1.07 ± 0.35	0.86 ± 0.26	0.96 ± 0.37	0.28 ± 0.02
Quercetin	2.11 ± 0.72	1.44 ± 0.56	1.43 ± 0.64	1.86 ± 0.82
Kaempferol	1.25 ± 0.25	1.43 ± 0.45	1.30 ± 0.52	1.37 ± 0.63

Total flavonols	4.43 ± 0.97	3.73 ± 0.88	3.69 ± 1.18	3.29 ± 0.66
